# Deep learning for Alzheimer’s disease: advances in classification, segmentation, subtyping, and explainability

**DOI:** 10.1186/s12938-025-01482-6

**Published:** 2025-12-29

**Authors:** Mohammed Rizwan Shaikh, Andrew Jeyabose, R. Vijaya Arjunan

**Affiliations:** 1 Manipal Institute of Technology, Manipal Academy of Higher Education, Manipal, Karnataka India; 2 Department of Neurology, School of Medicine, University of North Carolina at Chapel Hill, Chapel Hill, United States

**Keywords:** Alzheimer’s disease, Deep learning, Medical image analysis, Disease subtyping, Explainable Artificial Intelligence (XAI)

## Abstract

Alzheimer’s disease (AD) poses urgent significant challenges for early detection and personalized prognostication. Deep learning (DL) is now regarded as a pivotal technology for extracting subtle imaging and non-imaging biomarkers; yet translating these advances into clinical practice demands a coherent framework. In this review, we first survey input modalities from structural and functional MRI to PET, genetic profiles, and cognitive tests and the key public cohorts that supply multimodal data. We then categorize DL architectures into three complementary pillars: (1) end-to-end classification networks for direct diagnosis; (2) multimodal fusion strategies that integrate heterogeneous biomarkers; and (3) automated segmentation pipelines for precise anatomical delineation. We also examine subtyping algorithms that uncover latent AD phenotypes via clustering and decision-tree models. In order to fill the gap between high-performance DL and real-world adoption, we detail explainable AI methods that render model decisions transparent, and we review performance benchmarks including accuracy, sensitivity/specificity, Dice and Jaccard indices to contextualize efficacy. Finally, we discuss clinical translation, covering prospective validation, workflow integration, and regulatory/privacy considerations, before outlining challenges and future directions such as data heterogeneity, interpretability–accuracy trade-offs, early/preclinical detection, and federated learning. Our roadmap highlights the interdisciplinary collaborations and technical innovations needed to deliver robust, trustworthy, and scalable DL-based tools for Alzheimer’s care.

## Introduction

**Fig. 1:**
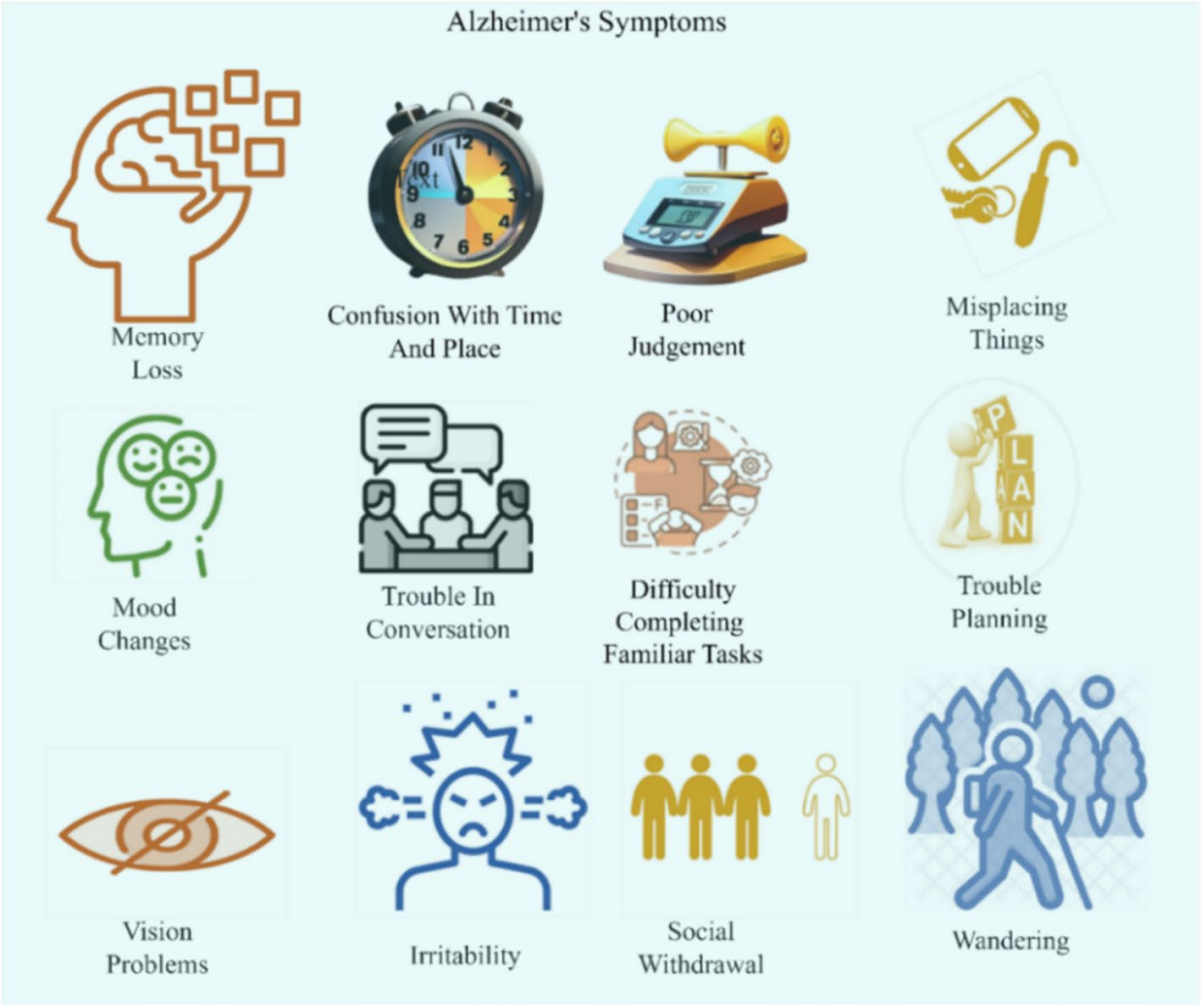
Early clinical manifestations of AD, including subtle cognitive decline (e.g., memory lapses), impaired judgment, difficulty performing familiar tasks, and changes in mood or personality

Alzheimer’s disease (AD) is the most common cause of dementia, an irreversible, progressive neurodegenerative illness marked by brain shrinkage, synapse loss, cell death that shows up as deficits in executive function, memory and language [1, 2]. Although there are various kinds of dementia, such as vascular dementia, dementia with Lewy bodies, and frontotemporal dementia yet AD accounts for roughly 60–70% of all cases [3]. Early identification of AD at the preclinical or mild cognitive impairment (MCI) stages is critical, as the disease process begins up to two decades before clinical symptoms emerge [4]. Figure 1 illustrates the subtle early signs such as occasional forgetfulness and slight executive difficulties that often go unnoticed until progression to more advanced stages.

**Fig. 2:**
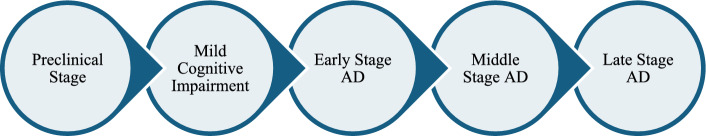
Overview of the progressive stages of AD, beginning with the preclinical stage, followed by MCI and advancing through early, moderate, and severe stages of dementia

AD typically advances through four clinically defined stages (preclinical, MCI, mild, moderate, and severe), each with escalating cognitive and functional decline (Fig. 2). In the preclinical phase, individuals remain asymptomatic despite underlying amyloid accumulation and neurofibrillary tangles. MCI represents a transitional state in which memory or other cognitive complaints arise without substantial interference in daily activities. When the illness develops into mild and moderate phases, deficits in planning, language, and spatial orientation become more pronounced; by the severe stage, patients lose autonomy in practically every aspect of daily life, and they frequently need constant attention [5].

**Fig. 3:**
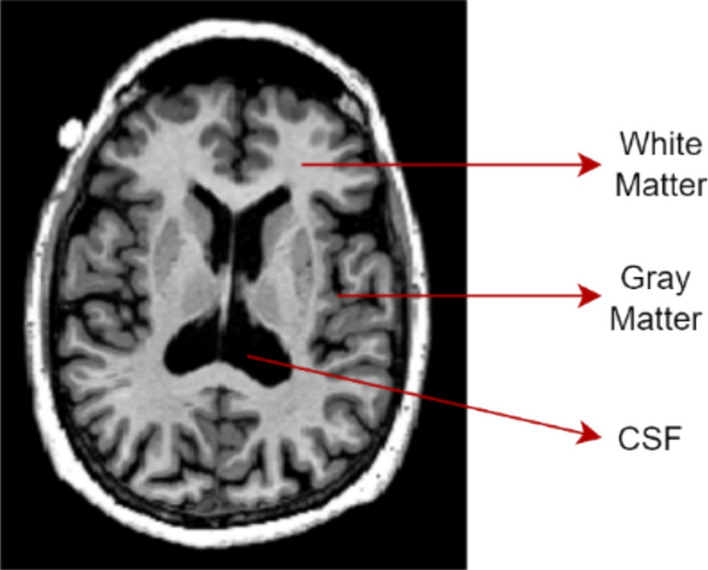
High-resolution T1-weighted MRI scan of the human brain illustrating detailed anatomical structure, including gray matter, white matter, and cerebrospinal fluid

**Fig. 4:**
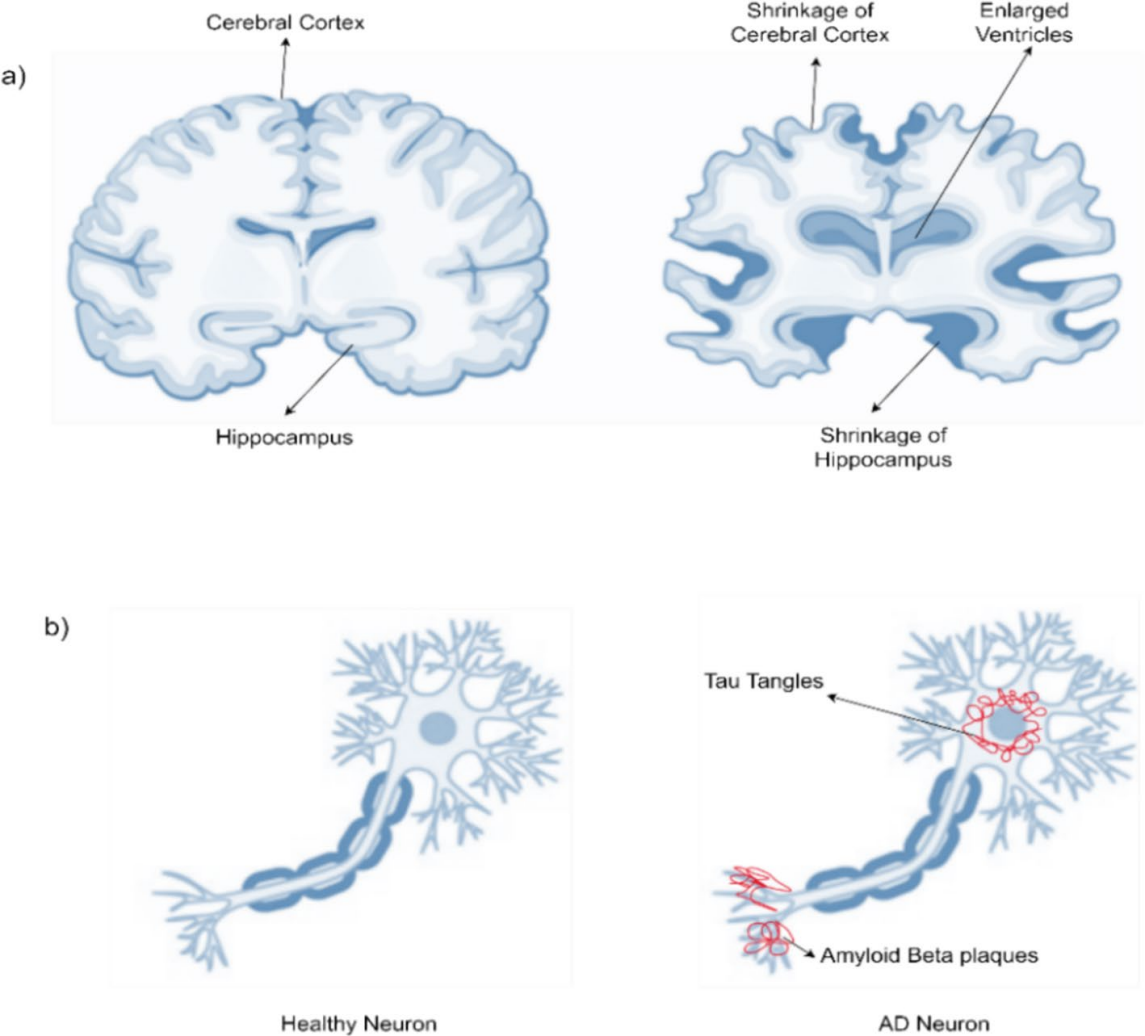
Images of brain and nerve cells. **a** Hippocampus, a key brain region involved in memory formation and one of the earliest affected areas in AD. **b** Structure of a neuron, illustrating deposit of amyloid β plaques and formation of tau tangles

The pathological features of AD include intracellular tau tangles, extracellular amyloid-β plaques, and extensive atrophy, particularly in the entorhinal cortex and hippocampal regions [6]. Cerebrospinal fluid (CSF) assays can detect amyloid and tau levels, while structural MRI reveals white matter (WM), gray matter (GM), and CSF volume changes (Fig. 3). Functional alterations can be captured via positron emission tomography (PET) or functional MRI (fMRI), highlighting disrupted connectivity and metabolic decline [7]. Figure 4 compares healthy hippocampal anatomy with that seen in AD, underscoring the importance of this region’s early atrophy [8].

Traditional diagnostic workflows—clinical assessments, CSF biomarkers, and neuroimaging—are resource-intensive and may lack sensitivity at the earliest stages. In recent years, DL models have demonstrated remarkable ability to learn subvisual patterns from large, multimodal datasets, enabling:

- Automated classification of AD versus healthy controls based on MRI/PET scans using convolutional neural networks (CNNs) [9],
- Longitudinal prognosis by modelling temporal disease trajectories with recurrent architectures (RNNs/LSTMs) [10],
- Data augmentation and latent representation learning via autoencoders and generative adversarial networks (GANs) to mitigate limited sample sizes [11],
- Multimodal fusion strategies that integrate imaging, genetic, and clinical features to improve diagnostic accuracy [12].

Yet, DL’s “black box” nature hinders clinical trust. Explainable AI (XAI) techniques like Grad-CAM, Integrated Gradients, SHAP and LIME are increasingly applied to highlight salient brain regions and validate model decisions against known AD pathology [13, 14]. Table 1 contrasts our review’s emphasis on multimodal DL architectures, XAI techniques, and pathways to clinical translation with prior surveys [7, 15–17].

**Table 1:** Comparison of the contributions of this review and other SOTA review papers

Contributions	Sarakshi et al 2022	Illakiya and Kathik 2023	Pallawi and Singh 2023	Sharma et al 2023	This paper
Non-imaging input modalities	✔	×	✔	×	✔
Prediction tasks	✔	✔	✔	×	✔
XAI	✔	×	×	×	✔
RNN	✔	✔	×	✔	✔
Performance evaluation metrics	×	✔	×	×	✔
Subtypes	×	×	×	×	✔
Up-to-date consensus available on AD diagnosis and prognosis	×	×	×	×	✔
Comparative analysis of the dataset	×	×	×	✔	✔
Segmentation	×	✔	×	×	✔

To ensure broad coverage of recent advances, we identified relevant papers through PubMed, Scopus, and Google Scholar using combinations of keywords such as “Alzheimer’s disease”, “deep learning”, “diagnosis”, “prognosis”, “subtypes”, and “explainable artificial intelligence”. To capture a diverse representation of methods, datasets, and perspectives, papers were carefully curated from the search results with emphasis on those that (1) proposed novel deep learning models for Alzheimer’s disease classification or prediction; (2) incorporated neuroimaging or clinical data; and (3) were published between 2019 and 2025 in peer-reviewed journals or conferences. This work follows a narrative review approach rather than a systematic review. While this allows for a flexible synthesis of rapidly evolving methodologies and concepts, it also introduces potential limitations such as selection bias and non-exhaustive inclusion of all studies. Nevertheless, considerable effort was made to ensure a balanced and comprehensive overview across imaging and non-imaging modalities, emphasizing methodological diversity and clinical relevance.

The layout of this paper is as follows: Sect. “Data sources and modalities” details available data sources and input modalities; Sect. “Deep-learning architectures for diagnosis” surveys DL architectures for AD classification, segmentation, and subtype analysis; Sect. “Explainability and model interpretation” reviews XAI methods for model interpretation; Sect. “Performance benchmarks and evaluation” presents performance benchmarks; Sect. “Clinical translation and deployment” explains translational efforts and real-world deployment; Sect. “Challenges and future directions” discusses current challenges and outlines future research directions; and Sect. “Conclusion” concludes with key take-home messages and a roadmap for next-generation DL systems in Alzheimer’s research and practice.

## Data sources and modalities

**Fig. 5:**
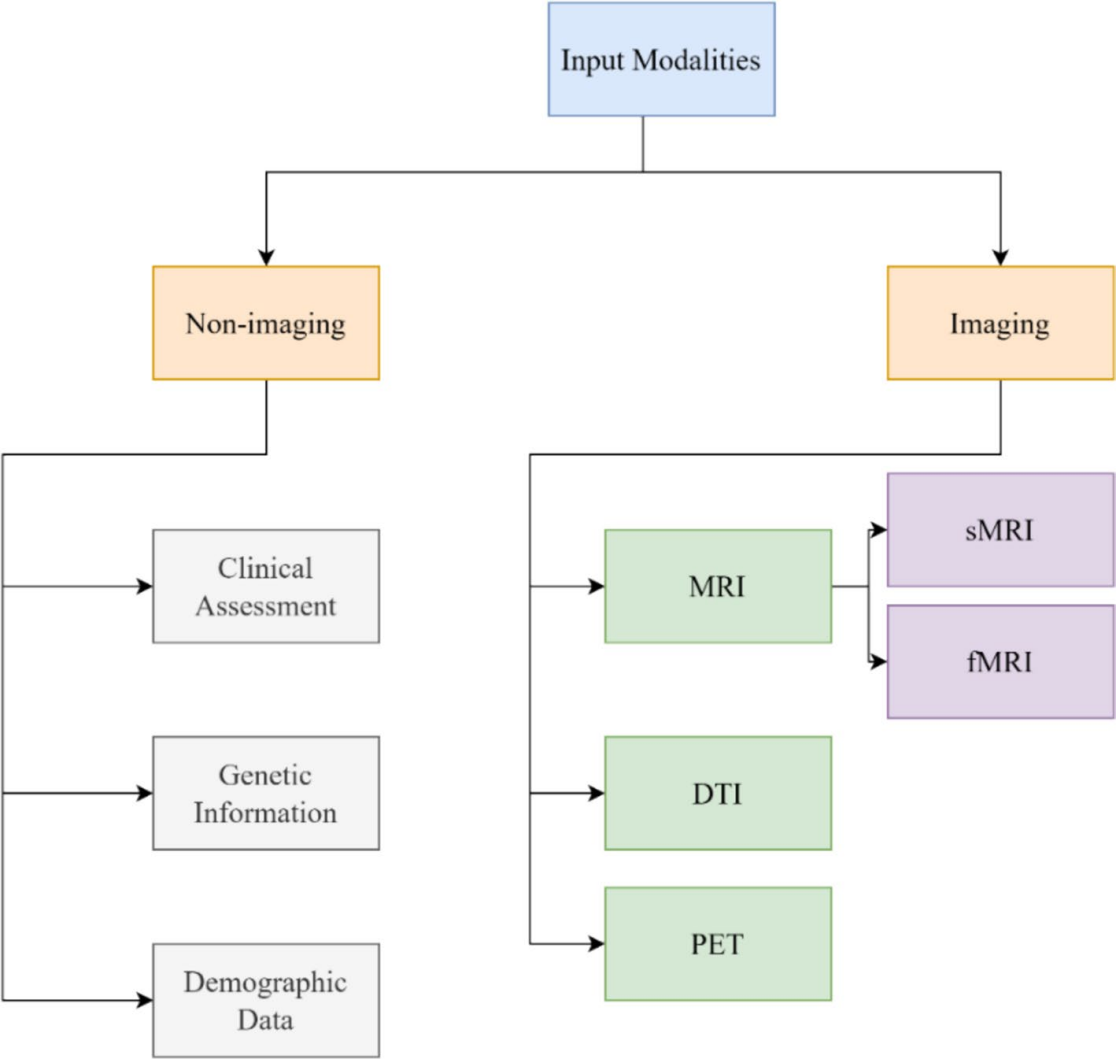
Overview of input modalities commonly used for AD diagnosis, including MRI, PET, DTI and other non-imaging data

Accurate Alzheimer’s diagnosis and prognosis increasingly depend on integrating multiple types of data, including high-resolution anatomical scans, molecular imaging, genetic profiles, and simple cognitive scores. Figure 5 illustrates how imaging and non-imaging inputs inform modern deep-learning pipelines. Below we describe each category in detail and then summarize the principal public cohorts that supply these multimodal data (Table 2).

**Table 2:** Overview of major public datasets offering multimodal data relevant to Alzheimer’s disease diagnosis and prognosis

Cohort	Approx. subjects	Modalities included	Key citation
ADNI	~ 2000	sMRI, fMRI, PET, CSF biomarkers, cognitive scores, genetics	[28]
OASIS	~ 1000	Serial sMRI, selective PET, cognitive assessments	[29–31]
AIBL	~ 1250	sMRI, amyloid PET, cognitive and lifestyle data	[32]
MIRIAD	~ 70	High frequency sMRI over two weeks to two years in AD and controls	[33]
NACC	~ 50,000	Standardized clinical, neuropathological, and genetic data across ADRCs	[34]

### Imaging

#### Structural MRI (sMRI)

Structural MRI produces three-dimensional volumetric maps of GM, WM, and CSF, enabling precise quantification of regional atrophy in Alzheimer’s disease. Early degeneration in the entorhinal cortex and hippocampus is readily captured by volumetric measures or cortical thickness analyses [18]. In deep-learning workflows, sMRI volumes may be pre-processed into two-dimensional slices (to reduce memory footprint), three-dimensional patches centred on key regions, or whole-brain inputs when sufficient GPU resources are available. Two-dimensional approaches streamline training at the cost of spatial context, whereas patch or volume-based methods retain anatomical fidelity but increase computational burden.

#### Functional MRI (fMRI)

Blood oxygen level-dependent (BOLD) contrast is used in fMRI to monitor neural activity and intrinsic connectivity among brain regions. Disruptions in the default mode and frontoparietal networks often precede gross atrophy, providing early signals of Alzheimer’s pathology [19]. Deep-learning pipelines typically either ingest raw time-series data, allowing recurrent or temporal-convolutional architectures to learn dynamic patterns, or convert signals into functional connectivity matrices that feed into graph-based or convolutional models. Both strategies leverage fMRI’s sensitivity to network dysfunction beyond structural change.

#### Diffusion MRI (DTI)

Diffusion tensor imaging characterizes the microstructural integrity of WM tracts by modelling the directionality of water diffusion. Measures like mean diffusivity and fractional anisotropy decline in tracts connecting memory-related regions, often before appreciable volume loss [20]. In machine-learning contexts, DTI data can be represented as voxel-wise scalar maps for three-dimensional convolutional networks or transformed into structural connectivity graphs that are input to graph neural networks, thereby highlighting tract-level disconnections indicative of early neurodegeneration.

#### Molecular PET imaging

Positron emission tomography employs radiotracers most commonly 18F-FDG for glucose metabolism, or amyloid and tau specific ligands to visualize molecular hallmarks of Alzheimer’s disease in vivo [21]. PET images, expressed as standardized uptake value ratios (SUVRs) or full volumetric reconstructions, serve as complementary inputs to structural and functional modalities. Three-dimensional convolutional architectures can jointly process PET and MRI volumes, exploiting metabolic and morphological contrasts to improve diagnostic accuracy and staging.

### Non-imaging

#### Cognitive and clinical assessments

**Fig. 6:**
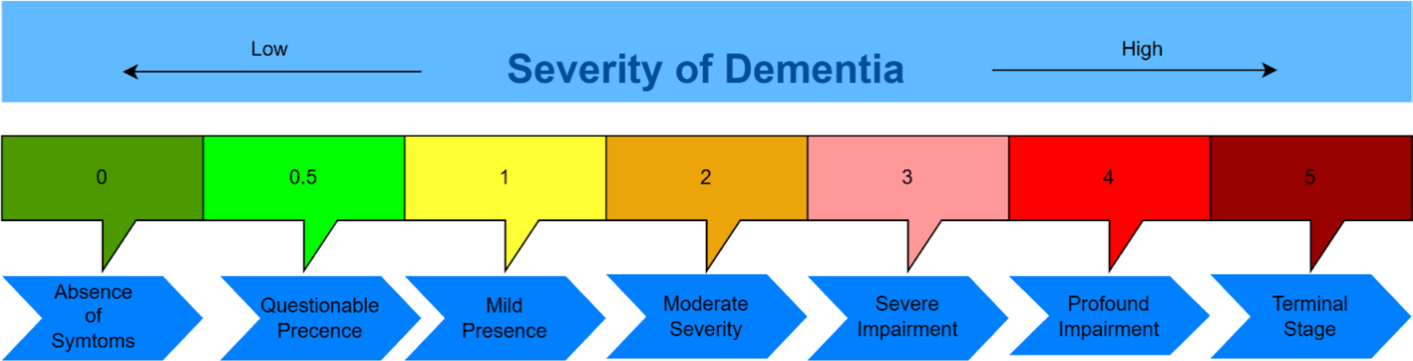
Clinical Dementia Rate Scale used to quantify the severity of symptoms in individuals with cognitive impairment

Standardized neuropsychological tests remain frontline tools in Alzheimer’s evaluation. The Mini-Mental State Examination (MMSE) is a 5–10 min, 30-point test, assessing orientation, recall, attention, language, and visuospatial skills; it flags global cognitive impairment but requires contextual interpretation based on age and education [22]. The Clinical Dementia Rating (CDR) scales six different functional areas like orientation, memory, judgment and problem-solving, personal care, home and hobbies, and community affairs on a 0–5 scale to yield a composite staging score (Fig. 6) [23, 24]. Longitudinal MMSE and CDR trajectories, combined with clinician and caregiver reports, provide temporal features that prognostic deep-learning models can forecast the shift from MCI to dementia.

#### Genetic risk factors

Genetic predisposition significantly modulates Alzheimer’s risk. The greatest prevalent risk mutation for late-onset AD is the apolipoprotein E gene (APOE) ε4 allele, roughly tripling risk in heterozygotes and up to 15-fold in homozygotes, although penetrance is incomplete [25]. Researchers are now developing polygenic risk scores that incorporate dozens of additional loci identified through genome-wide association studies, aiming to refine individual risk stratification. In deep-learning frameworks, genetic variants are typically encoded as categorical inputs or embeddings and fused with imaging and clinical features.

#### Demographics and lifestyle

**Fig. 7:**
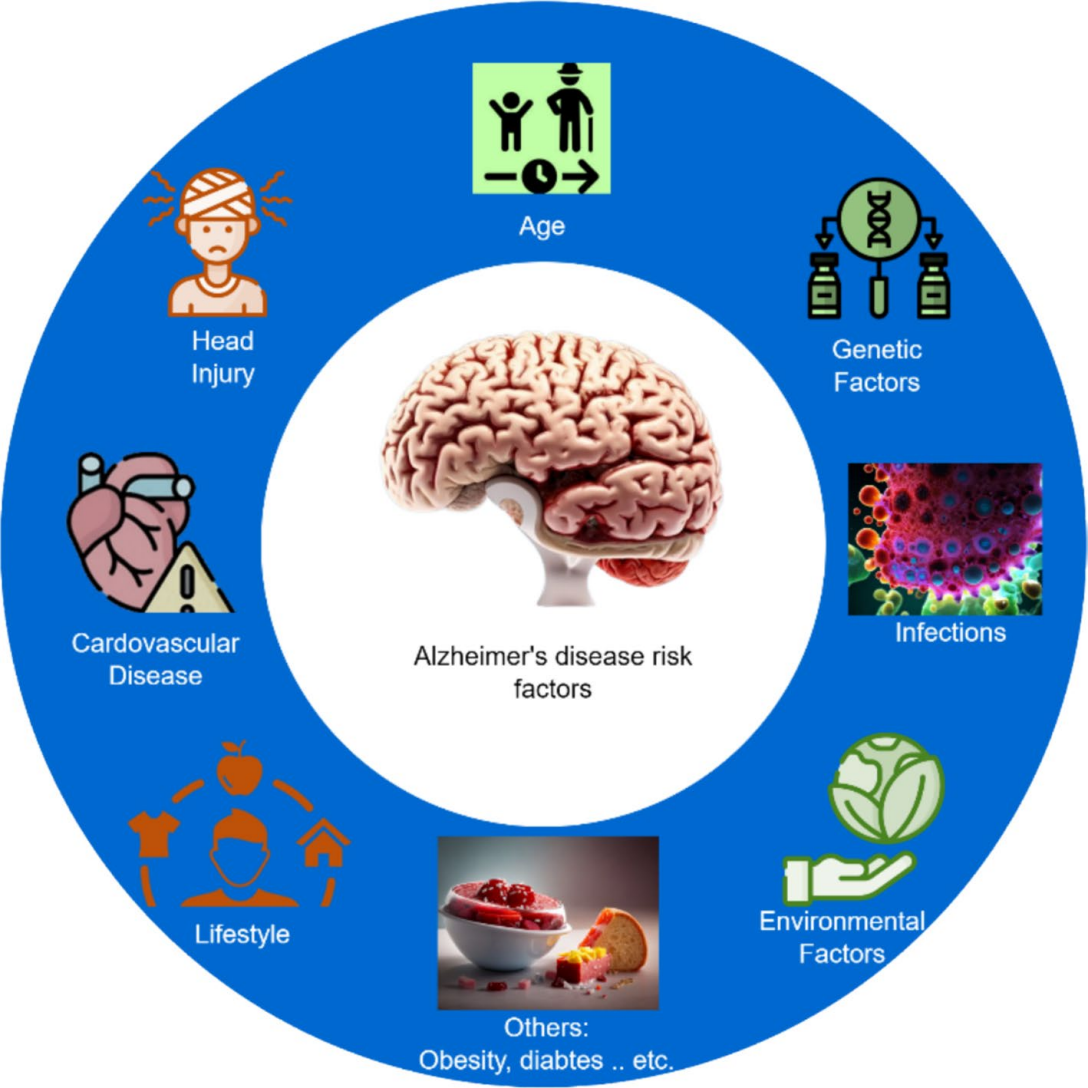
Illustration of various risk factors that elevate the risk of developing AD

Demographic variables and lifestyle factors further shape Alzheimer’s onset and progression. Incidence and rate of decline increase dramatically after age 65; while women experience higher lifetime incidence, some evidence suggests men decline more rapidly once symptomatic [26]. Educational attainment is a proxy for cognitive reserve, with higher education delaying clinical manifestations despite comparable pathology [27]. Socioeconomic status, physical activity, and diet also influence risk and resilience. These variables are often incorporated as tabular data, normalized or categorical, and merged with other modalities in multimodal deep-learning architectures (Fig. 7).

### Key public cohorts

In Alzheimer’s research large, well-annotated cohorts underpin the training and validation of deep-learning models. The Alzheimer’s Disease Neuroimaging Initiative (ADNI) includes roughly 2000 participants who contribute longitudinal sMRI, fMRI, PET, cerebrospinal fluid biomarkers, cognitive assessments, and genotyping data [28]. The OASIS cohort covers nearly 1000 subjects with serial sMRI scans and selected PET imaging alongside comprehensive neuropsychological evaluations [29–31]. Australia’s AIBL study, with more than 1250 older adults, provides amyloid PET, MRI, cognitive scores, and lifestyle data over more than 8500 person-years of follow-up [32]. The MIRIAD dataset, though smaller at approximately 70 individuals, offers densely sampled sMRI scans spanning 2 weeks to 2 years in both Alzheimer’s and control groups, facilitating precise atrophy-rate estimation [33]. Finally, the National Alzheimer’s Coordinating Center (NACC) aggregates standardized clinical, neuropathological, and genetic data from over 50,000 participants across 42 US research centres [34].

## Deep-learning architectures for diagnosis

**Fig. 8:**
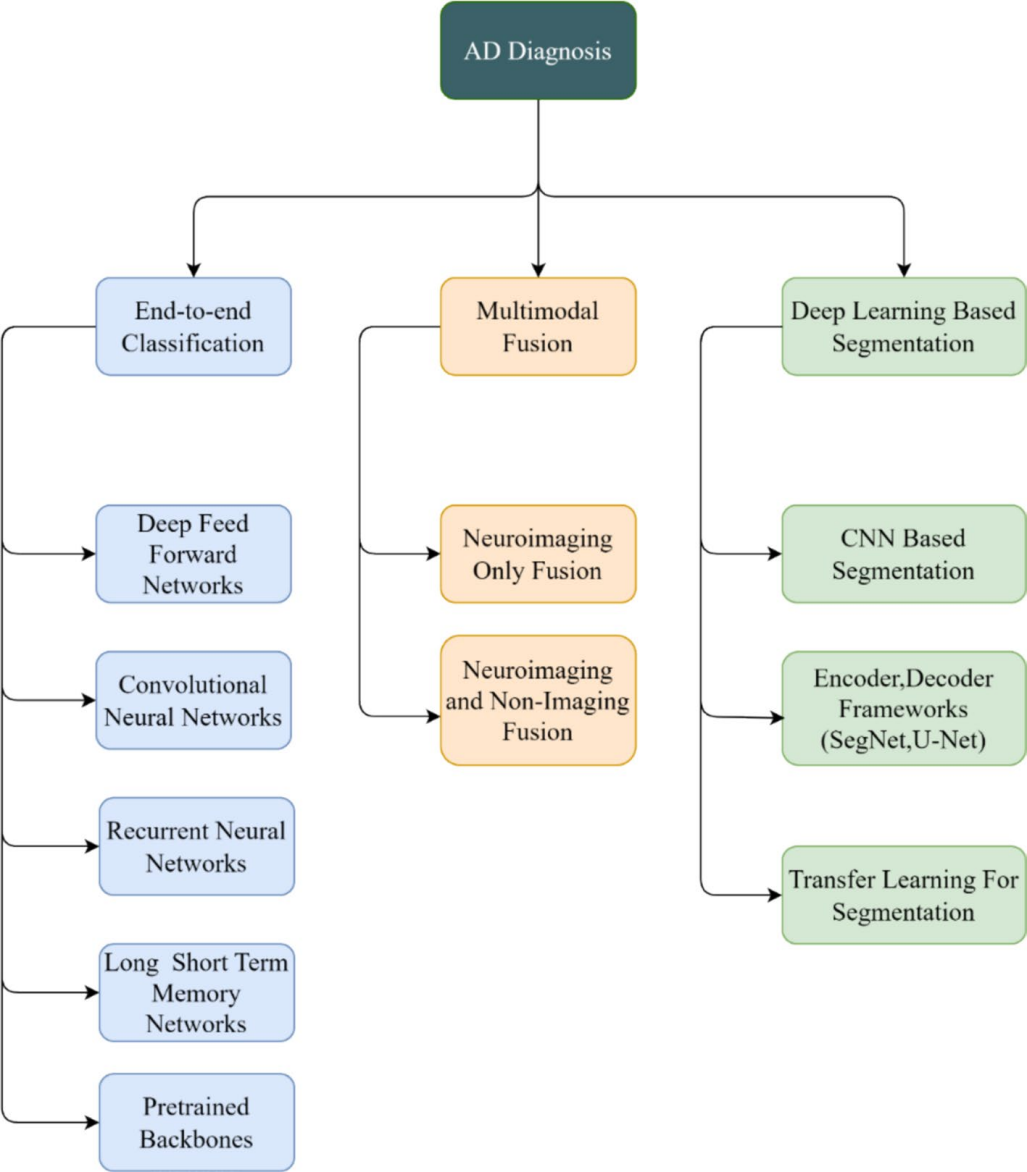
Representative deep learning architectures employed in the diagnosis of AD

Modern Alzheimer’s research leverages a spectrum of deep-learning architectures tailored to the heterogeneous, multimodal nature of disease markers. Figure 8 illustrates representative network designs, ranging from purely convolutional classifiers to hybrid generative and graph-based frameworks. In this section, we describe three principal categories: end-to-end classification networks, multimodal fusion strategies, and specialized frameworks for data augmentation and advanced relational modelling.

### End-to-end classification

End-to-end classifiers directly map raw or minimally pre-processed inputs to diagnostic labels, learning feature hierarchies without manual feature engineering. Table 3 provides the practical comparison of end-to-end architectures used in AD neuroimaging, detailing their typical inputs, strengths, weaknesses, and best use cases.

**Table 3:** Comparison of end-to-end architectures for AD neuroimaging analysis

Method	Typical input	Strengths	Weakness	Best use cases
MLP	Features (segmented + handcrafted)	Simpler, low data needs	Misses spatial context, needs careful engineering	Small datasets, engineered variables
2D CNN	MRI slices	Parameter efficient, faster	Ignores 3D anatomy, may miss patterns	Slice-wise analysis, small data
3D CNN	MRI/ PET volumes	Captures full 3D patterns	Slow, data hungry, risk of overfit	Multisite, larger cohorts
RNN / LSTM	Longitudinal data	Models temporal evolution	Needs multi-timepoint data, complex	Progression, MCI → AD studies
Pretrained models	Any image volume	Fast convergence, generalizes	May transfer artefacts, domain mismatch	Low cohort studies, rapid prototyping

#### Deep feedforward networks

Multilayer perceptrons (MLPs) represent some of the earliest applications of deep learning in Alzheimer’s research. By combining handcrafted features with fully connected layers, these models demonstrated the feasibility of applying neural networks to neuroimaging data [35]. Although they do not explicitly capture spatial structure, their simplicity and interpretability provided an important foundation for later, more specialized models.

#### Convolutional neural networks (CNNs)

CNNs have become the cornerstone of AD neuroimaging studies due to their ability to directly learn spatial representations from MRI and PET data [36–38]. 2D CNNs offer computational efficiency and are well-suited for smaller datasets, while 3D CNNs preserve volumetric structure and capture full-brain patterns of atrophy. Ensemble approaches further enhance robustness by combining multiple architectures, highlighting the versatility of CNNs across diverse applications [39, 40].

#### Recurrent neural networks (RNNs)

RNNs extend the capabilities of deep learning by modelling temporal dynamics in longitudinal data [41]. They are particularly valuable for studying disease progression and predicting conversion from MCI to AD, as they can capture sequential dependencies in imaging or clinical measures. While training RNNs can be challenging, their application to AD highlights the growing recognition of temporal patterns as critical for prognosis.

#### Long short-term memory networks (LSTMs)

LSTMs address many of the limitations of traditional RNNs through memory cells that retain long-term information [42]. When applied to resting-state fMRI and multimodal sequences, LSTMs have demonstrated strong predictive performance, especially in forecasting MCI-to-AD conversion. The integration of attention mechanisms with LSTMs has further enhanced their ability to focus on disease-relevant regions and timepoints, making them a powerful tool for prognostic modelling.

#### Pretrained backbones

Transfer learning with pretrained backbones such as VGG, ResNet, and DenseNet has accelerated progress in AD classification [43, 44]. By leveraging features learned from large-scale image datasets, these models converge faster and often generalize better to small medical cohorts. Increasingly, pretrained models are being adapted with contrastive and multi-task learning strategies, demonstrating their flexibility and potential to bridge gaps between limited medical data and high-capacity deep learning frameworks.

### Multimodal fusion

Multimodal fusion exploits the complementary strengths of different data types—structural and functional neuroimaging, cognitive tests, genetic markers, and clinical variables—to boost Alzheimer’s classification and prognosis [43, 45, 46]. By integrating heterogeneous sources, these models overcome the limitations of any single modality and capture richer disease signatures.

#### Neuroimaging-only fusion

Several studies have explored multimodal fusion of MRI, PET, and fMRI for AD classification as shown in Table 4. These approaches generally report higher accuracy compared to unimodal baselines, highlighting the complementary value of combining structural, functional, and metabolic information. The models employed range from modality-specific encoders and correlation-based alignment [47, 48] to wavelet-based image fusion with deep CNN classifiers [49], and even generative models that impute missing modalities, reflecting increasing technical sophistication in the field. Reported accuracies above 90% (e.g., Rallabandi et al. [49]) are promising but would benefit from validation on external cohorts to confirm robustness. Innovative approaches such as Gao et al. [50], which introduced GAN-based PET synthesis to mitigate missing data and domain shift, point toward creative strategies to address data limitations, though further work is needed to establish the clinical reliability of synthetic modalities.

**Table 4:** Summarizes key deep-learning models trained on multimodal neuroimaging inputs

Ref	Dataset	Input modality	No. of subjects	Prediction type	Performance metrics	Validation and notes
[47]	ADNI	sMRI, fMRI	120(AD-34, eMCI-18, lMCI, NC-50)	AD vs NC, AD vs MCI, MCI vs NC, LMCI vs EMCI	AD vs NC (Global brain) Accuracy = 88.0% Sensitivity = 80.0% Specificity = 93.3% Precision = 88.9% Recall = 80.0% F1-score = 84.2% AUC = 88.7%	Train/test Strength: joint MRI-fMRI features Limitation: small sample size, no external validation Maturity: preliminary
[48]	ADNI	sMRI, fMRI	145(AD-34, SMC-26, MCI-35, NC-50)	NC vs MCI, NC vs SMC, SMC vs MCI, NC vs AD, SMC vs AD, MCI vs AD,	AD vs NC Accuracy = 92.0% AUC = 90%	Train/test Strength: effective feature extraction Limitation: modest sample size, single dataset Maturity: exploratory
[49]	OASIS	T1 sMRI, PET	1098 (Over 2000 MRI, 1500 PET) 605 HC, 493 demented	MCI vs AD	Accuracy = 94.1%	Train/test/Val Strength: large dataset Limitation: lacks external validation Maturity: promising but dataset-specific
				HC vs MCI	Accuracy = 95.5%	
				HC vs AD	Accuracy = 95.9%	
				Overall	Accuracy = 95.06% Sensitivity = 97.41% Specificity = 92.76% AUC = 0.985	
[50]	ADNI I, ADNI II,	T1 MRI, PET	ADNI I 821(MRI) 326(PET)	pMCI vs sMCI	Accuracy = 75.3% Specificity = 74.1% AUC = 78.6% Sensitivity = 77.3% FIS = 69.9%	Cross dataset validation (Trained on ADNI1 and evaluated on ADNI 2) Strength: robust validation Limitation: Synthetic PET reliability uncertain Maturity: emerging
			ADNI II 534(MRI) 487(PET)	AD vs CN	Accuracy = 92.0% Specificity = 94.0% AUC = 95.6% Sensitivity = 89.1% FIS = 90.5%	

ADNI I and ADNI II refer to the first two phases of the ADNI. ADNI I (2004–2009) enrolled CN, MCI and mild-AD participants using 1.5 T MRI, FDG-PET, CSF biomarkers, and genetic data. ADNI II (2011–2016) expanded enrolment (CN, SMC, EMCI, LMCI, mild AD), upgraded to 3 T MRI, added amyloid PET, and broadened biofluid and cognitive tests

#### Neuroimaging and non-imaging fusion

Hybrid approaches that integrate neuroimaging with non-imaging information such as cognitive scores, genetic markers, and demographics have introduced diverse methodological strategies for AD prediction as shown in Table 5. Graph-based models leverage phenotypic similarities between subjects to guide representation learning [51], while attention mechanisms and transformer networks aim to capture higher-order dependencies between imaging features and non-imaging variables [52]. Other studies have explored imputation techniques to address missing modalities [53] or employed stacked autoencoders and gradient-boosting classifiers for late fusion of heterogeneous data types [54, 55]. These methodological innovations highlight the potential of hybrid models to move beyond unimodal learning and towards holistic disease characterization.

**Table 5:** Compares multimodal deep-learning frameworks that blend neuroimaging with clinical, genetic, and cognitive data

Ref	Dataset	Input modality	No. of subjects	Prediction type	Performance metrics	Validation approach and notes
[51]	ADNI I, ADNI II	FDG-PET, sMRI	461	AD vs CN	Accuracy = 96.68% Sensitivity = 99.19% Specificity = 94.49%	Train/test/Val Strength: Strong AD vs CN accuracy Limitation: weaker pMCI prediction Maturity: promising but dataset-limited
			331	sMCI vs pMCI	Accuracy = 78% Sensitivity = 54.96% Specificity = 89.37%	
[52]	ADNI I, ADNI II	sMRI, age	1355	pMCI vs sMCI	Accuracy = 73.6% Sensitivity = 59.1% Specificity = 82.1% AUC = 73.7%	Cross dataset validation (Trained on ADNI1 and evaluated on ADNI 2) Strength: robust AD vs CN performance Limitation: pMCI vs sMCI remains difficult Maturity: emerging
				AD vs NC	Accuracy = 90.5% Sensitivity = 84.6% Specificity = 95.0% AUC = 93.9%	
[53]	ADNI	MRI, PET, SNP	452	NC vs sMCI	Accuracy = 93.11%	Cross validation Strength: SNP integration enhances performance Limitation: Only internal validation Maturity: exploratory
			412	NC vs AD	Accuracy = 98.22%	
			393	NC vs pMCI	Accuracy = 97.35%	
[54]	ADNI I,II, GO	Clinical data, cross-sectional MRI, Genetic	3315 (SNP- 808), (MRI- 503), Neurological test data-2004)	CN vs MCI vs AD	EHR + SNP + Imaging Accuracy = 78% Precision = 77% Recall = 78% F1 Score = 78%	Cross validation Strength:very large multimodal dataset Limitation: no external validation Maturity: emerging
[55]	AIBL, ADNI, FHS, LBDSU, OASIS, PPMI, NIFD, NACC	MRI, demo- graphics, functional assessments, medical history, neuropsychological test results	–	NC vs MCI/DE	COG Accuracy = 0.804 ± 0.011 F-1 = 0.772 ± 0.012 Sensitivity = 0.771 ± 0.013 Specificity = 0.895 ± 0.006 MCC = 0.670 ± 0.018	External validation across 7 cohorts Strength: broad generalizability Limitation: performance modest vs single datasets Maturity: well validated

Nevertheless, several challenges remain. Graph formulations and deep attention mechanisms offer strong representational capacity, but they are often sensitive to hyperparameter tuning and can overfit in small-sample settings [51, 52]. Imputation-based methods reduce missing-data issues but risk introducing synthetic biases that may weaken biological interpretability [53]. Similarly, late fusion approaches are simple to implement but may fail to fully exploit cross-modal interactions [54, 55]. A further limitation is that most fusion pipelines prioritize accuracy over interpretability, leaving clinicians with limited insight into how non-imaging features influence predictions.

### Deep learning-based segmentation

Accurate delineation of key brain structures most notably the hippocampus and cortical regions is vital both for extracting volumetric biomarkers and for focusing downstream diagnostic models. Deep-learning segmentation methods automate this process, learning relevant patterns directly from MRI or PET images to yield precise anatomical masks with minimal manual intervention [56]. A summary of DL-based segmentation studies, including the dataset, study category, participant age, gender distribution, number of scans, and model architecture is presented in Table 6. Table 7 complements this by detailing dataset characteristics, outlining input modalities, number of subjects, prediction task types, and evaluation metrics, highlighting methodological variations across studies.

**Table 6:** Summary of deep learning segmentation studies, detailing dataset, study category, participant age, gender distribution, number of scans, and model architecture

Ref	Dataset	Category	Age (years)	Gender (M/F)	Scans#	Model used
[57]	ADNI	AD	75.9 ± 6.8	48/49	–	Multitask deep CNN + 3D DenseNet
		NC	75.9 ± 5.0	59/60		
		MCI	75.2 ± 7.3	145/88		
[58]	ADNI	CN MCI AD	–	71–96 61–96 55–93	637 548 635	CNN with inception
	OASIS	CN MCI AD		42–94 69–82 66–85	50 35 30	
[59]	ADNI	CN	71–86 M 77.3 ± 4 71–88 F 78.8 ± 4.8	40/40	80	SegNet, ResNet-101
		MCI	67–87 M 78.03 ± 5.8 65–82 F 73.63 ± 5	40/40	80	
		AD	72–92 M 79.03 ± 5.47 70–91 F 79.8 ± 6.98	40/40	80	
[60]	EADC—ADNI HarP	Normal	60–90	16/13	100	CNN
		MCI		13/8		
		LMCI		7/6		
		AD		20/17		
	MICCAI	–	19–34 (Training) 18–90 (Testing)	–	–	
[61]	ADNI	–	–	–	100	Transfer learning Spatial warping Network segmentation
	University hospital Modena	MCI			31	
	OCSAE hospital Modena	MCI			30	
	John Redcliff hospital Oxford UK	AD			12	

^*^MICCAI—The MICCAI dataset comprises 35 atlas scans and was initially utilized in the MICCAI 2012 Grand Challenge focussed on multi-atlas labelling

**Table 7:** Overview of datasets used in reviewed studies, including input modalities, number of subjects, prediction task types, and corresponding performance metrics

Ref	Dataset	Input modality	No. of subjects	Prediction type	Performance metrics
[57]	ADNI	sMRI	449	Hippocampus segmentation	Dice similarity coefficient = 87.0% PPV (positive predicted value) = 84.6% SEN_S (sensitivity) = 89.7% VE (volume error) = 6.0%
				MCI vs NC	Accuracy = 76.2% AUC = 77.5%
				AD vs NC	Accuracy = 88.9% AUC = 92.5%
[58]	ADNI	sMRI	349	Gray matter segmentation using EICA	
				ADNI AD vs MCI CN vs AD CN vs MCI CN vs MCI vs AD	Accuracy = 93.24% Accuracy = 100% Accuracy = 98.73% Accuracy = 95.61%
	OASIS		28	OASIS CN vs AD CN vs MCI AD vs MCI CN vs MCI vs AD	Accuracy = 92% Accuracy = 88.23% Accuracy = 91.76% Accuracy = 81.48%
[62]	Indira Gandhi Medical College Hospital	MRI	36	Segmentation	AD vs CN Accuracy = 99.7%
[59]	ADNI	sMRI	240	Segmentation of gyri and sulci contour, GM, WM, cortex thickness, cortex surface, CSF and hippocampus	Classification Accuracy = 95% Sensitivity = 96% Specificity = 93.9%
[60]	EADC-ADNI HarP	MRI (1.5 and 3.0 T)	100	Hippocampus Segmentation	EADC-ADNI HarP Mean Dice value = 0.9015 MICCAI Mean Dice value = 0.8835
	MICCAI	MRI (1.5 T)			
[61]	ADNI	MRI	-	Segmentation	Best model Mean dice = 0.878 ± 0.003 *P *value < 0.001
	University hospital Modena	MRI (3 T)			
	OCSAE hospital Modena	MRI (3 T)			
	John Redcliff hospital Oxford UK	MRI (3 T)			

#### CNN-based segmentation

Convolutional neural networks remain the backbone of many segmentation pipelines. Multitask CNNs that jointly segment structures such as the hippocampus while performing disease classification have demonstrated the feasibility of end-to-end pipelines [57]. Other work has focused on optimizing slice selection [58] or incorporating asymmetry-sensitive features to better capture hippocampal degeneration [62]. While these approaches improve diagnostic sensitivity, they are often tailored to narrow anatomical targets and may not generalize well across cohorts with variable imaging protocols. Moreover, reliance on handcrafted pre-processing steps (e.g., slice selection or asymmetry masks) can reduce model flexibility compared to fully automated frameworks.

#### Encoder–decoder frameworks (SegNet, U-Net)

Encoder–decoder architectures such as SegNet and U-Net are widely used for hippocampal and subfield segmentation because their skip connections preserve spatial detail [59, 63]. Variants that fine-tune U-Net hyperparameters (e.g., SHPT-Net) or integrate ResNet blocks (e.g., RESU-Net) have reported improved robustness and subfield-level accuracy. These frameworks benefit from architectural efficiency and strong spatial localization, but their performance is still heavily dependent on the quantity and quality of manual annotations.

#### Transfer learning for segmentation

When annotated data are scarce, transfer learning boosts performance by fine-tuning encoders pretrained on large natural-image datasets (e.g., ImageNet) [60, 61]. Ensemble strategies across multiple slice orientations [60] and domain adaptation of pretrained hippocampal segmenters [61] have shown accuracy comparable to expert raters, underscoring the utility of reusing pretrained weights. However, transfer learning approaches may import biases from non-medical source datasets, and their effectiveness diminishes when target data differ substantially in contrast or resolution.

### Deep-learning for Alzheimer’s disease subtyping

AD exhibits pronounced heterogeneity in its clinical course and underlying biology, motivating the use of subtyping methods to identify patient subgroups with shared progression patterns, genetic risk factors, or neuroimaging signatures. By uncovering these latent subpopulations, clinicians can personalize prognoses, tailor interventions, and design more informative clinical trials. Deep-learning algorithms have been used on both unimodal and multimodal datasets spanning structural MRI, functional MRI, PET, genetic markers, and cognitive assessments to derive robust and reproducible AD subtypes [64].

#### Clustering-based subtyping

Unsupervised clustering methods aim to reveal natural heterogeneity in AD without requiring predefined labels. Studies combining multimodal features from MRI, fMRI, and PET have identified stable subgroups with distinct functional disruptions, atrophy trajectories, or metabolic signatures [65–67]. Recent extensions incorporate clinical outcomes into clustering, enabling earlier detection of high-risk patients [68], while mixture-of-expert models have linked cortical and subcortical atrophy patterns with differential cognitive decline [69]. These approaches highlight the potential of clustering to uncover biologically meaningful subtypes, yet they also face challenges: cluster solutions are often dataset-specific, sensitive to parameter choices, and difficult to replicate across independent cohorts. Moreover, their clinical utility is limited by a lack of consensus on the number and stability of AD subtypes, and the interpretability of latent groupings remains an open issue.

#### Decision tree-based subtyping

Decision tree-based models provide a complementary, more interpretable route to AD stratification. By combining multimodal biomarkers and imaging-derived features, these methods generate explicit rules for subgroup assignment [70, 71]. Such transparency can facilitate clinical adoption by clarifying which features drive subtype differentiation. However, most decision-tree applications to date have been developed on relatively small cohorts, and their generalizability to diverse populations has not been firmly established.

#### Biological validity and clinical translation

Although deep learning-based subtyping has revealed meaningful imaging and cognitive patterns in AD, the biological relevance of these subtypes is often unclear. Most studies lack validation against neuropathological markers such as amyloid tau, limiting their interpretability. Similarly, few approaches demonstrate consistent clinical utility beyond statistical clustering-generalizability across cohorts and practical impact on patient management remain underexplored. Decision tree models offer more transparent stratification but are rarely validated in prospective or real-world settings. To enhance clinical translation, future work must align subtypes with known pathology, ensure reproducibility, and evaluate their usefulness in guiding diagnosis, prognosis, or treatment selection.

### Technical comparison and practical guidelines for model selection in AD research

Deep learning models used in AD research vary in structure and capabilities. For studies with limited sample sizes or computational resources, 2D CNNs, MLPs, or pretrained models are suitable due to their lower complexity and faster convergence, with pretrained models offering improved generalization through transfer learning [35–37]. When full-brain spatial context is essential such as in sMRI or PET analysis 3D CNNs are preferred, as they preserve volumetric anatomical information [43]. For longitudinal studies aiming to model disease progression, particularly MCI-to-AD conversion, RNNs or LSTMs are advantageous for capturing temporal dependencies [41, 42]. In settings where both imaging and tabular clinical data are available, hybrid architectures that combine CNNs with MLPs can effectively integrate heterogeneous information [72]. For multi-site datasets where scanner variability introduces domain shift, domain-adapted or adversarial models improve robustness. Additionally, for multimodal neuroimaging tasks involving MRI and PET, fusion-based models, especially 3D convolutions are recommended to capture inter-modal interactions [50].

### Foundation models for Alzheimer’s disease diagnosis

Foundation models (FM) are large-scale, pretrained deep learning models that have emerged as transformative tools in medical artificial intelligence, including AD diagnosis. Unlike traditional AI models, which are trained for specific tasks and data types, foundation models are developed using vast multimodal datasets and self-supervised learning, enabling them to generalize across various downstream applications in medicine [73]. For instance, AD Found integrates multimodal neuroimaging data using masked autoencoders and contrastive learning to capture intra and inter-modal relationships, though it faces challenges such as computational demand and the need for prospective validation [74]. Similarly, the LEAD foundation model for EEG-based AD detection demonstrates improved performance by addressing inter-subject variability through contrastive pretraining on the large EEG-AD dataset assembled, yet its clinical generalizability and methodological novelty invite further scrutiny [75]. Moreover, foundation models applied to clinical text and electronic health records, including large language models like Llama-3.1 and transformers for risk prediction, show potential to zero and few-shot learning, though concerns related to data bias, interpretability, and integration into clinical workflows remain prominent.

#### Computational requirements

Training and deploying FMs is computationally intensive, often involving hundreds of millions to billions of parameters and necessitating multi-node GPU/TPU clusters, high-memory accelerators, and distributed training frameworks [76]. Large-scale medical FMs like RadFM and COSMOS1050K required millions of radiology images and significant infrastructure to achieve robustness [77]. Even parameter-efficient tuning methods such as LoRA reduce costs but remain resource-demanding, limiting accessibility for smaller AD research groups [78].

#### Data needs and curation challenges

AD-specific multimodal datasets remain relatively small compared to natural image corpora. Resources like ADNI provide valuable data but lack the scale and diversity required for true FM level pretraining. Furthermore, privacy regulations, heterogeneous imaging protocols, and incomplete clinical annotations hinder centralized dataset creation. Recent large-scale multimodal datasets such as COSMOS1050K provide templates for future efforts, but neurodegenerative disease-specific collections are urgently needed.

#### Validation challenges and the clinical gap

Despite impressive zero-shot and few-shot performance, FMs face hurdles in real-world validation. Performance often declines when applied across scanners, demographics, or disease stages without domain adaptation. Moreover, there is no consensus on standardized benchmarks for FM evaluation in AD research. The black-box nature of FMs complicates interpretability, and regulatory frameworks are not yet designed for adaptive, prompt-based systems. This highlights the gap between the promises of FMs and their current clinical reality.

#### Addressing and exacerbating existing problems in AD diagnosis

Foundation models hold the potential to alleviate several persistent challenges in AD diagnosis. By leveraging large-scale pretraining and few-shot learning, they can help overcome the scarcity of labelled datasets, while their capacity for multimodal integration makes them well-suited for combining heterogeneous inputs such as MRI, PET, EEG, and clinical records within a unified framework. Moreover, their adaptability and continual learning capabilities provide avenues to mitigate domain shifts that typically arise in multi-site studies. At the same time, however, foundation models may also intensify existing problems. If training datasets underrepresent minority populations or diverse imaging protocols, they risk propagating and amplifying bias. Similarly, their high computation demands may exacerbate resource inequities, favouring large research centres while limiting access for smaller institutions. Finally, their inherent complexity raises interpretability concerns, potentially deepening the “black box” problem and hindering clinical trust and adaptation.

## Explainability and model interpretation

Because deep learning models can uncover intricate patterns from neuroimaging and multimodal datasets, their inclusion in AD research has accelerated recently. However, the opaque nature of these models poses a challenge for clinical adoption, where understanding why a model makes a specific decision is as crucial as the decision itself. Explainable Artificial Intelligence (XAI) offers a means to interpret and validate model outputs, providing transparency and enabling trust among clinicians and researchers. The term XAI is an established abbreviation for “Explainable Artificial Intelligence”, following standard scientific naming conventions where “X” denotes “explainable”. In the context of AD diagnosis and prognosis, XAI not only supports model transparency, but also plays a significant role in biomarker identification, clinical validation, and personalized treatment planning.

### Role of explainable AI in AD diagnosis

Explainable AI approaches provide substantial understanding into how deep learning and machine-learning models reason internally, which is particularly important in a healthcare context. In Alzheimer’s research, these techniques allow models to be interpreted by visualizing relevant anatomical regions, assessing risk factors, and validating the clinical plausibility of predictions. For instance, visualization tools such as heatmaps or attention maps help identify brain regions or genetic markers that significantly influence classification outcomes. This allows researchers to assess whether the AI model is focusing on areas of known clinical relevance, such as the temporal lobe or hippocampus, which are commonly affected in AD.

**Fig. 9:**
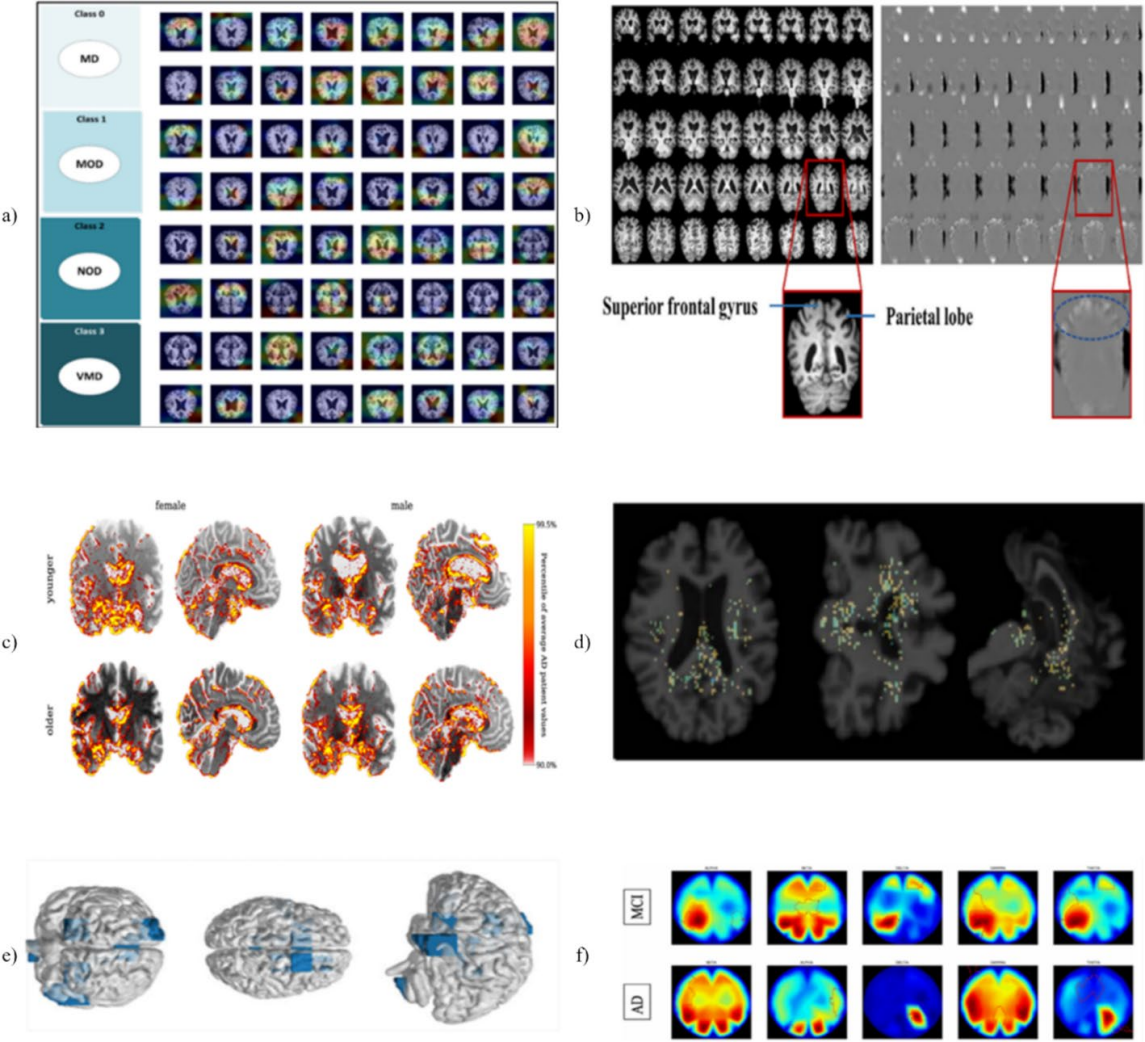
Sample outputs of various XAI techniques applied to AD diagnosis. **a** Grad-CAM [79], **b** Guided Grad-CAM [80], **c** layerwise relevance propagation [81], **d** integrated gradients [82], **e** Shapley Additive Explanations [83], **f** local interpretable model-agnostic explanations [84]

Moreover, XAI enables model developers and clinicians to identify the most distinctive characteristics contributing to disease progression or subtype classification. Through this, one can trace the model’s decision-making process and ensure alignment with established clinical knowledge. XAI also assists in identifying potential biases in the training dataset, verifying fairness across demographic groups, and supporting hypothesis generation for novel biomarkers. Additionally, the capacity to clarify the model decisions allows for increased clinician reliance and smoother integration into existing clinical workflows, thereby promoting the practical use of AI-assisted diagnostic tools in real-world healthcare systems. Validating interpretability and transparency in XAI remains a multidimensional challenge, particularly in the healthcare domain. Traditional performance metrics such as accuracy and AUC do not capture the quality or trustworthiness of explanations generated by explainable AI techniques. Therefore, alternative evaluation criteria are essential. Among these, fidelity measures how accurately the explanation reflects the model’s internal decision-making process; sparsity assesses whether the explanation is concise and highlights only the most relevant features; and human-grounded evaluation involves comparing the explanation with expert knowledge or clinical expectations to determine its plausibility. Common validation strategies include expert assessments to evaluate clinical relevance and comparisons with established domain knowledge to confirm that the explanations align with known biomarkers of Alzheimer’s disease. Ultimately, a combination of technical and human-centered evaluation approaches is crucial for establishing trust in XAI systems used in medical applications. Figure 9 illustrates representative examples of visual explanation results, while Table 8 summarizes deep learning models in Alzheimer’s disease research that incorporate explainable techniques, and Table 9 provides an overview of six widely used XAI methods along with their evaluation strategies.

**Table 8:** An overview of studies that improve the interpretability and transparency of deep learning models in AD diagnosis by utilizing XAI techniques

Ref	Dataset	Input modality	No. of subjects	Prediction type	Performance metrics	XAI technique	XAI evaluation criteria
[79]	Kaggle	3D MRI		MD vs MOD vs NOD vs VMD	Accuracy = 97.05%, AUC = 99.89%, F1-score = 97.05%, Precision = 97%, Recall = 97%	Grad-CAM	Fidelity
[80]	ADNI	MRI	212 (AD-98, NC-114)	AD vs NC	Accuracy = 92.68% Sensitivity = 89.47% Specificity = 95.45% Precision = 94.44% F1 = 91.89% G-mean = 92.41%	Guided Grad-CAM	Human-grounded evaluation
[81]	ADNI	3D sMRI	432 (AD-306, HC-126) 1254 images (593 Women + 661 Men)	HC vs AD	Balanced Accuracy (Women) = 87.58 ± 1.14%, (Men) = 79.05 ± 1.27%, Sensitivity Women = 83.77 ± 2.34%, Men = 71.10 ± 2.13%, Specificity Women = 91.38 ± 0.97%, Men = 86.99 ± 1.29%	LRP	Fidelity
[86]	ADNI I, ADNI II, ADNI III	T1 MRI	502 (CN 252, AD 250)	AD vs CN	Model B with 44 channels Accuracy = 87.25%	IG	Human-grounded evaluation
[83]	ADNI I, II, III	sMRI	1193	AD vs NC	ACC = 0.920 ± 0.088, SEN = 0.920 ± 0.119, SPE = 0.919 ± 0.052, AUC = 0.967 ± 0.023	SHAP	Sparsity Sparsity
				pMCI vs sMCI	ACC = 0.819 ± 0.044, SEN = 0.818 ± 0.055, SPE = 0.816 ± 0.056, AUC = 0.857 ± 0.029		
[87]	Kaggle OASIS NCBI	MRI, Genetic data	–	MD vs MOD vs ND vs VMD	CNN Avg accuracy = 97.3% Avg precision = 97.2% Avg recall = 98.1% Avg F MEASURE = 97.3%	LIME	Human-grounded evaluation
					SVC Accuracy = 82.4% Precision = 81.8% Recall = 81.7% F measure = 82.2%		

^*^MD mild demented, *MOD* moderate demented, *NOD* non-demented, *VMD* very mild demented

**Table 9:** Overview of six widely used XAI techniques, highlighting their model compatibility, interpretability type, strengths, and limitations

Technique	Model type	Interpretability type	Strengths	Limitations
Grad-CAM	CNNs	Local, visual	Class-discriminative heatmaps for images	Limited to convolutional layers; coarse outputs
Guided Grad-CAM	CNNs	Local, visual	Combines fine-grain and class-specific info	Not applicable to non-CNN models
LRP	DNNs	Local, feature-based	Layer-wise relevance propagation rules	Requires layer specific configurations
IG	Any differentiable model	Local, feature-based	Baseline-based integration of gradients	Sensitive to choice of baseline
SHAP	Any model	Local and global, feature-based	Strong theoretical foundation	Computationally expensive for large models
LIME	Any model	Local, feature-based	Simple, model-agnostic, intuitive explanations	Instability due to random perturbations

### Visual explanation techniques

To improve the interpretability of deep learning models used for AD diagnosis, a number of visual explanation techniques have been put forth. These methods vary in their implementation and underlying principles but share a common goal: to provide meaningful, interpretable outputs that align with human understanding and medical expertise.

#### Grad-CAM

Gradient-weighted Class Activation Mapping (Grad-CAM) is a well-known technique for visualizing CNN outputs that explain model predictions by highlighting influential image regions [85]. Its strength lies in intuitive heatmap overlays that allow researchers to link network focus with anatomical regions. In AD applications, such as ADD-Net [79], Grad-CAM has facilitated interpretability by pinpointing pathology-associated areas in sMRI. However, the method’s reliance on coarse, last-layer activations often yields diffuse maps that may lack the spatial precision needed for fine-grained neuroanatomical interpretation.

#### Guided Grad-CAM

Guided Grad-CAM combines Grad-CAM’s localization with pixel-level gradients, producing sharper and more class-specific explanations [88]. This was leveraged in 3D HA-ResUNet for MCI subtype detection [80]. While effective in producing high-resolution voxel-level maps, the technique inherits the instability of gradient-based methods, raising concerns about reproducibility across scanners and pre-processing pipeline.

#### Layerwise relevance propagation (LRP)

Layerwise relevance propagation redistributes a prediction back to input features, enforcing conservation of total relevance. Its voxel-level attributions have been used to investigate bias in AD models [81]. LRP’s strength lies in offering mathematically grounded, faithful relevance maps; however, results can be highly sensitive to the choice of propagation rules.

#### Integrated gradients (IG)

Integrated gradients is an attribution technique that measures how much each input feature contributes to a prediction by computing the integral of gradients along a path from a baseline input to the actual input [89]. This method addresses the shortcomings of gradient-based approaches by producing more stable and consistent explanations. In comparative analyses [86], IG generated coherent heatmaps aligned with known AD biomarkers, outperforming Grad-CAM and LRP in stability.

#### SHapley additive explanations (SHAP)

SHAP, grounded in cooperative game theory, quantifies feature contributions globally and locally [90]. In patch-based AD studies [83], SHAP ranked MRI-derived features by their predictive value, enhancing both performance and interpretability. While SHAP offers robust theoretical grounding, its computational cost can be prohibitive in high-dimensional neuroimaging data.

#### Local interpretable model-agnostic explanations (LIME)

LIME approximates complex models with simple local surrogates to explain individual predictions. Applied to multimodal AD studies [87], LIME clarified contributions of both imaging and genomic features. Its appeal lies in model-agnosticism and case-specific insights, but explanations can vary depending on perturbation strategy, limiting consistency across runs.

LIME was used to explain gene-level contributions to the classification decisions.

### Challenges and practical considerations in XAI for AD

While XAI techniques such as Grad-CAM, LIME, SHAP, Integrated Gradients, and LRP are increasingly used in AD-related research, several practical challenges limit their clinical impact:

1. Instability and reproducibility issues

Many XAI techniques are sensitive to model architecture, input noise, and training variations. For example, Grad-CAM outputs may shift significantly even with small changes in weights or input pre-processing. This undermines trust, especially in safety-critical domains in healthcare.

2) Risk of misleading explanations

Saliency maps and feature attributions may highlight regions that correlate with the prediction but are causally relevant. Overreliance on heatmaps may lead to incorrect biological interpretations.

3) Low clinical interpretability

XAI outputs are often not aligned with how clinicians interpret brain imaging or assess symptoms. There is a gap between algorithmic explanations and domain-relevant reasoning. Few studies involve neurologists or radiologists in the evaluation of explanation quality.

4) Lack of standard evaluation metrics

There is no consensus on how to measure the quality of explanations. Metrics such as localization accuracy, fidelity, completeness, and clinician trust are rarely reported. This hinders objective comparison of XAI techniques.

## Performance benchmarks and evaluation

The effectiveness of the proposed system for Alzheimer’s disease (AD) classification and hippocampal segmentation was quantitatively assessed using standard evaluation metrics. These measures reflect the model’s effectiveness in distinguishing between AD and non-AD subjects, as well as the accuracy of anatomical segmentation outputs. This section presents both classification and segmentation metrics, along with the mathematical formulations used for evaluation.

### Evaluation metrics for classification

To assess the classification performance, researchers use a set of standard metrics derived from four outcomes:

- True positive (TP): AD patients correctly classified as AD.
- True negative (TN): healthy individuals correctly recognized as non-AD.
- False Positive (FP): healthy individuals mistakenly identified as AD.
- False negative (FN): AD patients incorrectly classified as cognitively normal.

Below is a breakdown of the measures used to assess the model’s diagnostic potential: accuracy represents how many cases, out of all samples were classified correctly:

1 $${\text{Accuracy }} = \,\,\frac{{\left( {{\text{TP}} + {\text{TN}}} \right)}}{{\left( {{\text{TP + TN + FP + FN}}} \right)}}$$

Sensitivity (recall) indicates the proportion of actual AD patients that a model correctly classifies:

2 $${\text{Sensitivity }} = \,\,\frac{{\left( {{\text{TP}}} \right)}}{{\left( {{\text{TP + FN}}} \right)}}$$

Specificity indicates the model’s ability to avoid falsely labelling healthy individuals as having AD:

3 $${\text{Specificity }} = \,\,\frac{{\left( {{\text{TN}}} \right)}}{{\left( {{\text{TN + FP}}} \right)}}$$

Precision assesses how many of the subjects labelled as AD by the model are actually AD patients:

4 $${\text{Precision = }}\frac{{\left( {{\text{TP}}} \right)}}{{\left( {{\text{TP + FP}}} \right)}}$$

F1-score indicates the model’s ability to detect both positive and negative cases of AD properly:

5 $${\text{F1 score = 2*}}\frac{{\left( {{\text{Precision*SEN}}} \right)}}{{\left( {{\text{Precision + SEN}}} \right)}}$$

Cohen’s kappa is a metric that determines how reliably two individuals classify items into categories, accounting for chance agreement:

6 $${\text{k}}\,{\text{ = }}\,{\text{2*}}\,\frac{{\left( {{\text{TP*TN - FN*FP}}} \right)}}{{\left( {{\text{TP + FP}}} \right){\text{*}}\left( {{\text{FP + TN}}} \right){\text{ + (TP + FN)*}}\left( {{\text{FN + TN}}} \right)}}$$

### Evaluation metrics for segmentation

Segmentation metrics are crucial for evaluating the spatial accuracy of anatomical structures such as the hippocampus. The segmentation network’s effectiveness is evaluated using the following metrics:

Dice similarity coefficient (DSC): it measures the degree of spatial agreement between the predicted segmentation and the ground truth:

7 $$DI= \frac{\left(2\text{TP}\right)}{\left(2\text{TP}+\text{FP}+\text{FN}\right)}.$$

Jaccard index (JI): like the Dice index [91], quantifies how much two sets intersect relative to their union:

8 $${\text{JI = }}\frac{{\left( {{\text{TP}}} \right)}}{{\left( {{\text{TP + FP + FN}}} \right)}}$$

where

- TP represents true positivity, refers to correctly identified hippocampal voxels that fall within the actual positive regions of the reference standard.
- FP represents false positivity, refers to incorrectly identified hippocampal voxels that fall outside the actual positive regions of the reference standard.
- FN represents false negativity, refers to background voxels that were missed despite being located within the actual positive regions of the reference standard.

Hausdorff distance: the Hausdorff distance [92] gives the farthest distance between any point in the predicted segmentation and its closest corresponding point in the ground truth segmentation and vice versa. It tests the greatest deviation between the predicted and ground truth segmentations. It represents the proportion of pixels in the segmentation result that are absent in the ground truth segmentations. The formula for the Hausdorff distance between two points A and B is given by:

9 $${\text{H(A,B) = max(}}\left( {{\text{h}}\left( {{\text{A,B}}} \right){\text{,h}}\left( {{\text{B,A}}} \right)} \right)$$

where $$h\left( {A,B} \right) = \max {\text{ }}\begin{array}{*{20}c} {} \\ {a \in Ab \in B} \\ \end{array} \min \left| {\left| {a - b} \right|} \right|$$

Precision: precision quantifies how accurate the segmentation model is when making positive predictions:

10 $${\text{Precision = }}\frac{{\left| {{\text{X}} \cap {\text{Y}}} \right|}}{{\left| {\text{Y}} \right|}}$$

Recall: this metric quantifies the ability of the segmentation model to correctly detect all relevant voxels of the hippocampus:

11 $${\text{Recall = }}\frac{{\left| {{\text{X}} \cap {\text{Y}}} \right|}}{{\left| {\text{X}} \right|}}$$

where X denotes the collection of voxels classified as hippocampal tissue by the proposed approach and Y denotes the equivalent collection of voxels in the reference standard mask.

### Evaluation metrics for subtyping

To evaluate the quality and clinical significance of AD subtyping approaches, researchers typically employ a comprehensive validation framework that incorporates both supervised clustering metrics and supervised classification measures. The assessment of clustering solutions commonly utilizes the silhouette coefficient to quantify how well individual patients are assigned to their respective subtypes by measuring the ratio of intra-cluster cohesion to inter-cluster separation. Complementarily, the Davies–Bouldin index provides insight into cluster quality by evaluating the average tightness and separation of identified subtypes, with lower values indicating better-defined groupings:

12 $${\text{Silhouette = }}\frac{{\text{1}}}{{\text{N}}}\sum\nolimits_{{{\text{i = 1}}}}^{{\text{N}}} {\frac{{{\text{b(i) - a(i)}}}}{{{\text{max}}\left\{ {{\text{a(i),b(i)}}} \right\}}}}$$

where a(i) = average distance from point i to all other points in the same cluster. b(i) = minimum average distance from point i to all points in the nearest cluster:

13 $$DB{\text{ }} = {\text{ }}\frac{1}{N}\sum\nolimits_{{i = 1}}^{K} {\mathop {\max }\limits_{{j \ne i}} } \left( {\frac{{\sigma _{i} + \sigma _{j} }}{{d\left( {c_{i} ,c_{j} } \right)}}} \right)$$

where k = number of clusters; σ_i_ = average distance of all points in cluster i to their cluster centre; d (c_i_, c_j_) = distance between cluster centres i and j.

## Clinical translation and deployment

Bringing deep-learning tools for AD diagnosis from the research lab into routine care requires rigorous validation in real-world settings, seamless integration into clinical workflows, and robust compliance with regulatory and privacy standards. Below, we outline key considerations and cite recent evidence where these steps have been successfully demonstrated.

### Validation in prospective cohorts

Retrospective performance on public datasets, while necessary, is not sufficient for clinical adoption. Prospective multi-site validation demonstrates that a model retains accuracy across different patient populations, imaging hardware, and protocols. For example, Wang et al. created a dual-interaction deep-learning technique combining sMRI, genetic data and clinical characteristics, that was tested on ADNI-2 (3 T) and trained on ADNI-1 (1.5 T), achieving consistent predictive accuracy for long-term MCI-to-AD conversion across both cohorts [93]. Such inter-scanner and inter-site validations are critical to ensure that performance does not degrade when deployed in new hospital settings. These prospective validations build confidence that deep-learning algorithms can generalize to everyday clinical populations. Similarly, other studies have validated deep learning models trained on one dataset (ADNI) and tested on external cohorts such as AIBL, demonstrating maintained accuracy (C-index up to 0.781) over follow-up periods as long as 6 years, and improving risk stratification by combining imaging baseline and clinical measures [94].

More recently, Qiu et al. developed a longitudinal deep learning model that predicts AD conversion up to 4 years before onset with 80% accuracy, showing strong correlations with clinical and biomarker profiles and generalizability across external cohorts [95]. Dyrba et al. demonstrated that 3D-CNNs trained on ADNI and validated on multiple independent cohorts, including AIBL, can reach high diagnostic accuracy (AUC 0.91 for AD vs CN). Using relevance mapping, they showed that model focus aligns with key pathological regions like the hippocampus, with a strong correlation to measured atrophy, thus enhancing interpretability and biological plausibility [96].

These prospective and external validations are critical, building confidence that deep learning algorithms can generalize to heterogeneous clinical populations and just too tightly controlled research cohorts. However, while promising, these studies still fall short of full clinical translation. Most rely on highly curated research cohorts (ADNI, AIBL) that do not capture the full heterogeneity of clinical practice, where comorbidities, incomplete data, and variable imaging protocols are common. Moreover, few studies include true prospective trials within hospital workflows, meaning performance in controlled research environments may overestimate clinical effectiveness.

### Integration with radiology workflows

Effective deployment demands embedding AI seamlessly into radiologists’ existing Picture Archiving and Communication Systems (PACS) and reporting tools. Automated segmentation and heatmap overlays produced by explainable AI modules can be delivered as DICOM-SR annotations, allowing radiologists to review model-highlighted regions directly alongside standard images. A recent narrative review describes how AI algorithms for dementia imaging can automatically extract connectivity metrics from resting-state fMRI and generate structured reports that integrate with PACS, reducing manual processing time by over 50% and improving inter-rater reliability [97].

Clinical radiology practices have begun pilot programs in which AI-driven alerts flag scans with suspected AD pathology, prompting radiologists to include dedicated hippocampal atrophy measurements in their reports. Early results indicate a 30% increase in detection rates of mild cognitive impairment, with no significant workflow slow-down, demonstrating that AI can augment rather than hinder radiologist efficiency.

Market leaders such as Philips, in partnership with icometrix, are already rolling out AI-enabled imaging solutions (e.g., icobrain ARIA) on new MRI systems, providing fully automated, quantitative diagnostic and monitoring outcomes for AD that are fully integrated with clinical informatics platforms and PACS [98].

### Regulatory considerations and data privacy

Regulatory approval for AI-based medical devices hinges on demonstrating safety, effectiveness, and reproducibility. Agencies such as the European Medicines Agency (EMA) and the U.S. Food and Drug Administration (FDA) and now offer clear frameworks for Software as a Medical Device (SaMD). Key requirements include thorough technical documentation, risk management plans, and real-world performance monitoring.

Data privacy regulations GDPR in Europe and HIPAA in the United States govern the handling of patient health information used both for training and deployment. The ADNI serves as a model for data governance, providing open-access imaging and clinical data under strict de-identification protocols while enabling broad scientific collaboration.

Moreover, a recent review of clinical translation in molecular imaging emphasizes the importance of implementing federated learning and on-device inference to mitigate privacy risks by keeping sensitive data on-premises and sharing only model updates or aggregated statistics with central servers [99]. Such architectures align with both regulatory expectations and institutional data governance policies, facilitating the secure deployment of AI across healthcare networks.

Yet in AD diagnosis specifically, no deep learning tools have yet achieved broad FDA or EMA approval, highlighting the immaturity of the field relative to oncology or cardiology. Privacy-preserving federated frameworks remain largely theoretical in AD imaging, with minimal real-world deployment across hospital networks. Regulatory uncertainty about how to evaluate adaptive, continuously updated models further widens the gap between research innovation and clinical implementation.

## Challenges and future directions

The promise of deep learning for AD diagnosis and prognosis is tempered by several scientific, technical, and practical challenges. Addressing these hurdles is essential to translate research advances into reliable clinical tools.

### Data heterogeneity and scarcity

Datasets for Alzheimer’s research frequently contain a small number of samples, heavily skewed toward a few large consortia (e.g., ADNI), and collected under varying protocols, scanner types, and demographic distributions. This heterogeneity undermines model generalization: a network trained on high-field 3 T research scans may perform poorly on lower-resolution clinical images. Moreover, the gradual progression and clinical variability of AD mean that fully annotated longitudinal data key for predicting conversion from mild cognitive impairment remain scarce. Expanding and harmonizing multi-centre repositories, adopting standardized pre-processing pipelines, and leveraging data-augmentation strategies (including generative adversarial networks) are imperative to build robust, generalizable models [66].

### Interpretability versus accuracy trade-offs

State-of-the-art deep networks achieve high classification accuracy by exploiting millions of parameters, yet their “black box” nature impedes clinical trust. Explainable AI techniques such as Grad-CAM, attention maps and Shapley value attributions offer insights into model reasoning but often come at the cost of added computational overhead and potential reductions in raw performance. Future work must strike a balance, developing architectures with built-in interpretability (e.g., attention-gated convolutional layers) that deliver both transparency and diagnostic accuracy. Rigorous user studies with radiologists and neurologists will be crucial to assess the clinical utility of different explanation methods [86].

### Moving toward early and preclinical detection

The pathological changes of AD begin decades before clinical symptoms emerge. Detecting these subtle preclinical signals such as small hippocampal subfield atrophy or network-level connectivity disruptions poses a major challenge for both data and algorithms. Deep models must be sensitive to these faint biomarkers while remaining robust to noise. Transfer learning from large, related datasets (e.g., normal aging cohorts), and self-supervised pretraining on unlabelled MRI or PET scans, represent promising avenues. Future research should also integrate fluid biomarkers (CSF, plasma tau/amyloid ratios) and digital phenotyping (passive smartphone monitoring) as early indicators, moving deep-learning diagnostics into the true preclinical window [4].

### Federated and privacy-preserving learning

Centralized data pooling is often infeasible due to patient-privacy regulations and institutional policies. Federated learning enables the training of global models on decentralized datasets; hospitals retain local data while sharing only model updates. Recent pilot studies in neuroimaging have demonstrated that federated convolutional networks can achieve performance on par with centrally trained models, without exposing patient-level data. Incorporating differential privacy and secure aggregation protocols will further safeguard sensitive information. Scaling these approaches across diverse healthcare systems is a key future direction to overcome data scarcity, reduce selection bias, and accelerate real-world deployment [52].

## Conclusion

Deep learning has ushered in a new era for Alzheimer’s disease diagnosis and prognosis, delivering rapid, data-driven insights that far exceed the capabilities of traditional methods. In this narrative review, we have highlighted three core technical pillars—end-to-end classification, precise anatomical segmentation, and robust subtyping—that together sharpen diagnostic accuracy and enable personalized treatment strategies. Explainable AI techniques connect sophisticated model results with clinical confidence by ensuring that predictions can be verified against established disease patterns. Pretrained backbone networks and advanced localization methods further accelerate development and enhance sensitivity to subtle disease markers, while multimodal fusion integrates structural, functional, molecular, and clinical data into a comprehensive disease profile.

Looking forward, the field must address persistent challenges in data heterogeneity, interpretability, and early/preclinical detection. Emerging foundation models like large-scale, self-supervised architectures trained on diverse biomedical data offer promising gains in transferability and multimodal learning but require further investigation into their clinical validity, transparency, and computational feasibility. Federated and privacy‐preserving learning frameworks offer promising solutions to scale model training across institutions without compromising patient confidentiality. To translate these advances into routine care, interdisciplinary collaboration uniting AI researchers, neurologists, radiologists, and ethicists will be essential. By combining methodological rigour with thoughtful deployment strategies, future deep-learning systems hold the potential to transform Alzheimer’s care through earlier diagnosis, more reliable prognostication, and truly personalized interventions.

## Data Availability

No datasets were generated or analysed during the current study.
